# Can heart rate variability parameters derived by a heart rate monitor differentiate between atrial fibrillation and sinus rhythm?

**DOI:** 10.1186/s12917-018-1650-6

**Published:** 2018-10-25

**Authors:** B. Broux, D. De Clercq, L. Vera, S. Ven, P. Deprez, A. Decloedt, G. van Loon

**Affiliations:** Equine Cardioteam, Department of Large Animal Internal Medicine, Faculty of Veterinary Medicine, Ghent University, Salisburylaan 133, 9820 Merelbeke, Belgium

**Keywords:** Arrhythmia, Artifact correction, Cardioversion, Equine

## Abstract

**Background:**

Heart rate variability (HRV) parameters, and especially RMSSD (root mean squared successive differences in RR interval), could distinguish atrial fibrillation (AF) from sinus rhythm(SR) in horses, as was demonstrated in a previous study. If heart rate monitors (HRM) automatically calculating RMSSD could also distinguish AF from SR, they would be useful for the monitoring of AF recurrence. The objective of the study was to assess whether RMSSD values obtained from a HRM can differentiate AF from SR in horses. Furthermore, the impact of artifact correction algorithms, integrated in the analyses software for HRV analyses was evaluated. Fourteen horses presented for AF treatment were simultaneously equipped with a HRM and an electrocardiogram (ECG). A two-minute recording at rest, walk and trot, before and after cardioversion, was obtained. RR intervals used were those determined automatically by the HRM and by the equine ECG analysis software, and those obtained after manual correction of QRS detection within the ECG software. RMSSD was calculated by the HRM software and by dedicated HRV software, using six different artifact filters. Statistical analysis was performed using the Wilcoxon signed-rank test and receiver operating curves.

**Results:**

The HRM, which applies a low level filter, produced high area under the curve (AUC) (> 0.9) and cut off values with high sensitivity and specificity. Similar results were obtained for the ECG, when low level artifact filtering was applied. When no artifact correction was used during trotting, an important decrease in AUC (0.75) occurred.

**Conclusion:**

In horses treated for AF, HRMs with automatic RMSSD calculations distinguish between AF and SR. Such devices might be a useful aid to monitor for AF recurrence in horses.

**Electronic supplementary material:**

The online version of this article (10.1186/s12917-018-1650-6) contains supplementary material, which is available to authorized users.

## Introduction

With a prevalence of 0.3–2.5% of the population, atrial fibrillation (AF) is the most common clinically important arrhythmia in horses. Treatment, either by pharmacological or electrical cardioversion, is often successful, but recurrence rates are reported to be relatively high (16–43%) [1–5]. Most horses with AF have little or no detectable underlying cardiac diseases and clinical signs limited to different grades of poor performance. During strenuous exercise horses with AF can reach extremely high heart rates which may lead to R-on-T phenomenon and, in rare cases, even collapse or sudden death [6–8]. Timely diagnosis and treatment are important. After successful AF treatment, home monitoring by means of frequent auscultation or ECG is advised to check for AF recurrence. However, this cannot always be performed by lay people, requiring frequent veterinary visits or specialized equipment, which is time consuming and expensive.

Heart rate variability (HRV) describes and quantifies the beat-to-beat variability and the long-term variation in heart rate [9]. The heart rate is influenced by both the autonomic nervous system and the neuroendocrine system. Cyclical and beat-to-beat variations in heart rate are normal in the healthy individual. In horses, HRV has been used to study the autonomic regulation of the heart and animal welfare, quantifying stress and pain [10–12]. Aside from its use as indicator of autonomic tone, HRV is used in human medicine to diagnose arrhythmias [13, 14]. Since arrhythmia, especially AF, leads to an increased beat-to-beat interval variation, HRV parameters describing short-term variability are increased compared to sinus rhythm (SR) and algorithms using HRV parameters are implemented in devices detecting AF in humans [15–19]. In horses, HRV has already been evaluated for the detection of AF in horses before and after transvenous electrical cardioversion [20]. In that study, 6 different HRV variables were calculated from RR intervals after beat-to-beat QRS identification on the ECG trace, both during AF and during SR. RMSSD, the root mean squared successive differences in RR interval, yielded the best results with high sensitivity and specificity to distinguish AF from SR.

Different types of heart rate monitors (HRM) are increasingly being used in horses and some of them offer basic HRV calculations, including RMSSD [11, 21, 22]. They might be an accessible diagnostic tool for both veterinarians and horse owners to monitor horses for AF recurrence. Most HRMs, however, have not been validated for use in horses and significant differences between their RR registration and RR intervals from ECG recordings have been shown [23]. Therefore the purpose of this study was to assess whether AF and SR can be differentiated in horses based upon RMSSD generated from a HRM or automatically analyzed ECG trace, using that from manually identified RR intervals from an ECG as gold standard. Furthermore the impact of artifact correction prior to HRV analysis was evaluated.

## Materials and methods

### Study design and study population

Fourteen warmblood horses were presented to the Faculty of Veterinary Medicine, Ghent University for the treatment of AF. All horses were client-owned horses admitted to our clinic for cardiac examination and treatment of AF. AF was confirmed in all horses by an experienced cardiologist using an ambulatory ECG recording. All horses were warmblood horses with a mean ± SD age, bodyweight and height at the withers of 12 ± 6 years, 544 ± 62 kg and 167 ± 13 cm. The study population consisted of 5 mares, 8 geldings and 1 stallion. All horses were included in the study with written informed owner consent and cared for according to the principles outlined in the NIH Guide for the Care and Use of Laboratory Animals. Each horse was simultaneously equipped with a HRM and an ECG and subjected to a recording at rest and a standardized exercise test including 5 min of walk and 10 min of trot, both in AF (before cardioversion) and in SR (5 days after cardioversion). The recordings of both devices was started at the same time. After the examinations and AF treatment, all horses returned home with the owners.

### Electrodes and heart rate detection

Three different systems of heart rate detection (electrodes + RR detection) were compared. A modified base apex ECG was recorded as described elsewhere [20], using adhesive electrodes (Skintact, Leonhard Lang GmbH, Innsbruck, Austria) and analyzed using a commercial ECG software program (Televet 100 software version 5.1.2, Engel Engineering Services GmbH, Heusenstamm, Germany) with a detection limit of 8%, meaning that all RR intervals differing more than 8% from the previous RR interval were detected as outliers, but included in the analysis. First, the RR intervals automatically detected by the ECG software, leaving errors in QRS detection in place, were exported (ECG_Aut_). Subsequently, all RR intervals were checked by one experienced observer and manually corrected within the software in case of artifacts or wrong QRS detection to obtain manually corrected RR intervals (ECG_Man_) which served as gold standard. Furthermore, a HRM using a commercial plastic electrode set (Polar equine heart rate electrode set, Polar Electro Benelux, Dendermonde, Belgium) and a heart rate sensor for RR detection (Polar heart rate sensor H7, Polar Electro Benelux, Dendermonde, Belgium) was attached according to the manufacturer’s instructions. Also for this detection method, errors in RR detection by the HRM were left in the database.

### Heart rate variability and artifact correction methods

From all 14 horses, RR intervals from both ECG_Man_ and ECG_Aut_ were imported in Kubios Software (Kubios Heart Rate Variability Analysis Software, Varsitie 22, 70,150 Kuopio, Finland) at default settings (smooth priors and Lambda 500) for HRV analysis, while RR data from the HRM were imported into its own commercial software program (Polar Flow Software, Polar Electro Benelux, Dendermonde, Belgium) (Table 1). Subsequently, a time-matched 2-min recording at rest, walk and trot, both in AF and SR, was selected for calculation of RMSSD using the following formula:

$$ \mathrm{RMSSD}=\sqrt{\frac{1}{N-1}\sum \limits_{j=1}^{N-1}{\left({\mathrm{RR}}_{j+1}-{\mathrm{RR}}_j\right)}^2}. $$

**Table 1:** Median and range of the RMSSD values of 14 horses before (in atrial fibrillation (AF)) and after cardioversion (in sinus rhythm (SR))

Pace	Method	Artifact correction	RMSSD AF		RMSSD SR		*P*-value
			Median	Range	Median	Range	
Rest	ECG_Man_	none	670	248–1409	85	26–378	0.024*
Rest	ECG_Aut_	none	674	244–1409	118	26–580	0.024*
Rest	ECG_Aut_	Kubios very low	216	194–293	87	26–181	.0.024*
Rest	ECG_Aut_	Kubios low	195	160–233	82	16–175	0.024*
Rest	ECG_Aut_	Kubios moderate	145	125–193	72	26–145	0.024*
Rest	ECG_Aut_	Kubios high	78	61–112	61	25–94	0,79
Rest	ECG_Aut_	Kubios very high	23	13–98	30	18–56	1
Rest	HRM	Polar Flow	233	72–329	78	32–169	0.024*
Walk	ECG_Man_	None	229	84–495	43	11–185	0.024*
Walk	ECG_Aut_	None	226	153–495	83	11–194	0.024*
Walk	ECG_Aut_	Kubios very low	189	124–236	34	11–113	0.024*
Walk	ECG_Aut_	Kubios low	163	117–219	34	11–104	0.024*
Walk	ECG_Aut_	Kubios moderate	135	105–161	34	11–79	0.024*
Walk	ECG_Aut_	Kubios high	82	62–102	28	11–63	0.024*
Walk	ECG_Aut_	Kubios very high	33	11–52	22	10–45	1
Walk	HRM	Polar Flow	126	49–167	32	6–73	0.024*
Trot	ECG_Man_	None	89	53–144	10	5–18	0.024*
Trot	ECG_Aut_	None	125	34–377	86	7–221	1
Trot	ECG_Aut_	Kubios very low	93	52–187	13	7–58	0.024*
Trot	ECG_Aut_	Kubios low	84	48–124	13	7–53	0.024*
Trot	ECG_Aut_	Kubios moderate	71	43–122	13	7–36	0.024*
Trot	ECG_Aut_	Kubios high	52	34–85	13	7–32	0.024*
Trot	ECG_Aut_	Kubios very high	22	14–49	11	5–32	0,22
Trot	HRM	Polar Flow	37	25–108	6	5–16	0.024*

Two-minute recordings at rest, walk and trot were analyzed using 4 different methods of RR detection and HRV analysis. The automatically analyzed ECG followed by beat-to-beat manual correction of QRS detection (ECG_Man_) with HRV analysis using Kubios HRV Software without artifact filter was considered the gold standard method. *HRM* Polar heart rate monitor (Equine H7) with plastic electrodes and Polar Flow Software. *ECG*_*Aut*_ ECG with automatic RR interval detection (Televet 100) and Kubios HRV software with 5 different artifact correction levels. The artifact correction algorithm identifies every RR interval that differs from the average RR interval of the 2-min recording more than a specified threshold value as artifacts and replaces the corrupted RR interval by interpolated RR values. The threshold values used in this study were 0.45 s (very low artifact correction), 0.35 s (low), 0.25 s (moderate), 0.15 s (high) and 0.05 s (very high). * Indicates significant differences

Each recording was obtained 2 min after a change in gait, in order to exclude movement artifacts and stress reactions from the gait change.

Furthermore, for the ECG_Aut_ method, within the Kubios HRV software program, 5 different levels of artifact correction were applied. The artifact correction algorithm of this program compares every RR interval value against the average RR interval of the 2-min recording. The average is obtained by median filtering of the RR interval time series, and is therefore not affected by single outliers in RR interval time series. RR intervals that differ from the local average more than a specified threshold value, are identified as artifacts and corrected by replacing them by interpolated RR values using a cubic spline interpolation. The threshold values used in this study were 0.45 s (very low artifact correction; values that differ more than 0.45 s from the average), 0.35 s (low), 0.25 s (moderate), 0.15 s (high) and 0.05 s (very high). For example, the “Medium” correction level will identify all RR intervals that are 0.25 s longer/shorter compared to the local average. Detailed information about filtering methods is free available online in the Kubios user manual. For the ECG_Man_ method, no artifact correction was applied, while the commercial software program used for the RR intervals obtained with the HRM method, uses its own, unknown artifact correction algorithm.

### Statistical analysis

Statistical analysis was performed using dedicated software (SPSS 24, IBM Analytics, Brussels, Belgium). Descriptive statistics, quantile-quantile plots and Shapiro-Wilk tests were used to check for normality of data.

Data were not normally distributed and therefore nonparametric related samples Wilcoxon signed-rank tests were used to detect significant differences in RMSSD between horses in AF and SR at different paces using the different detection methods and artifact correction levels. Bonferroni corrections were applied to account for multiple (× 24) comparisons. Receiver operating characteristic (ROC) curves were used to study the performance of each method and each artifact correction level as a discriminatory variable for the detection of AF. The coordinate points of the ROC curves were used to determine the most fitting cut off values for each parameter at each recording. Cut off values were chosen to maximize sensitivity whilst retaining good (≥80%) specificity, where possible.

## Results

Mean values for RMSSD are displayed in Table 1. RMSSD values obtained from the ECG were significantly different (*P* < 0.05) between horses in SR and horses in AF, except when a high level of artifact correction was used and at trot if no artifact correction was applied. RMSSD values obtained with the HRM were significantly different between horses in SR and horses in AF, at all paces.

AUCs, cut off values and their sensitivity and specificity for the use of RMSSD as a discriminatory variable for the detection of atrial fibrillation are displayed in Table 2. Using the Polar Flow software in combination with a plastic electrode set (HRM), led to reliable cut off values, especially during exercise. ECG_Aut_, without artifact correction, led to reliable results at rest and at walk (AUC 0.96), but there was an important decrease in AUCs during trotting exercise (AUC 0.75). Low level artifact correction during trotting exercise, led to a clear improvement in AUC (=1) and cut off values with very high sensitivity and specificity. High levels of artifact correction resulted in an important drop in AUCs, especially at rest. Receiver operating curves (ROC) are available as Additional file 1.

**Table 2:** Area under the curve (AUC), cut off values and their sensitivity and specificity, and 95% confidence intervals (95% CI) for the use of RMSSD as discriminatory variable to distinguish atrial fibrillation from sinus rhythm in 14 warmblood horses

Pace	Method	Artifact correction	AUC	cut off	sens		spec	
						95% CI		95% CI
Rest	ECG_Man_	none	0.99	215	1	0.77–1	1	0.77–1
Rest	ECG_Aut_	none	0.96	383	0.93	0.66–1	0.93	0.66–1
Rest	ECG_Aut_	1	1	188	1	0.77–1	1	0.77–1
Rest	ECG_Aut_	2	0.99	131	1	0.66–1	0.93	0.66–1
Rest	ECG_Aut_	3	0.94	111	1	0.77–1	0.93	0.66–1
Rest	ECG_Aut_	4	0.77	65	0.93	0.66–1	0.69	0.42–0.92
Rest	ECG_Aut_	5	0.37	24	0,5	0.23–0.77	0.38	0.13–0.65
Rest	HRM_Elec_	Polar Flow	0.93	136	0.86	0.57–0.98	0.86	0.57–0.98
Walk	ECG_Man_	none	0.97	121	0.93	0.66–1	0.93	0.66–1
Walk	ECG_Aut_	none	0.96	164	0.93	0.66–1	0,86	0.57–0.98
Walk	ECG_Aut_	1	1	119	1	0.77–1	1	0.77–1
Walk	ECG_Aut_	2	1	111	1	0.77–1	1	0.77–1
Walk	ECG_Aut_	3	1	92	1	0.77–1	1	0.77–1
Walk	ECG_Aut_	4	0.99	57	1	0.77–1	0.93	0.66–1
Walk	ECG_Aut_	5	0.74	22	0.78	0.49–0.95	0.57	0.29–0.82
Walk	HRM_Elec_	Polar Flow	0.97	70	0.93	0.66–1	0.93	0.66–1
Trot	ECG_Man_	none	1	36	1	0.77–1	1	0.77–1
Trot	ECG_Aut_	none	0.77	90	0.86	0.57–0.98	0.72	0.42–0.91
Trot	ECG_Aut_	1	1	51	1	0.77–1	0.93	0.66–1
Trot	ECG_Aut_	2	1	42	1	0.77–1	0.93	0.66–1
Trot	ECG_Aut_	3	1	40	1	0.77–1	1	0.77–1
Trot	ECG_Aut_	4	1	33	1	0.77–1	1	0.77–1
Trot	ECG_Aut_	5	0.93	15	0.93	0.66–1	0.93	0.66–1
Trot	HRM_Elec_	Polar Flow	1	21	1	0.77–1	1	0.77–1

Two-minute recordings at rest, walk and trot were analyzed using 4 different methods of RR detection and HRV analysis. The automatically analyzed ECG followed by beat-to-beat manual correction of QRS detection (ECG_Man_) with HRV analysis using Kubios HRV Software was considered the gold standard method. *HRM* Polar heart rate monitor (Equine H7) with plastic electrodes and Polar Flow Software. *ECG*_*Aut*_ ECG with automatic RR interval detection (Televet 100) and Kubios HRV software with 5 different artifact correction levels. The artifact correction algorithm identifies every RR interval that differs from the average RR interval of the 2-min recording more than a specified threshold value as artifacts and replaces the corrupted RR interval by interpolated RR values. The threshold values used in this study were 0.45 s (very low artifact correction), 0.35 s (low), 0.25 s (moderate), 0.15 s (high) and 0.05 s (very high)

## Discussion

The goal of our study was to assess an easily applicable tool for home monitoring of AF recurrence after treatment. In the current study pre-treatment AF could be differentiated from post-treatment SR based upon RMSSD using a commercially available HRM. Investigation of different artifact filtering levels showed that, compared to no filtering, low levels of artifact correction improved sensitivity and specificity, especially during trotting exercise. However, sensitivity and specificity fell with higher levels of filtering.

In this study RMSSD was chosen as the HRV parameter to distinguish AF from SR. RMSSD is a time domain HRV parameter and especially useful in assessing short term HRV [9, 24]. In a previous study, using 6 different HRV parameters for the differentiation between AF and SR in horses, RMSSD yielded the best results with cut off values with high sensitivity and specificity, identifying all cases of AF with only a small chance of false positives [20]. Furthermore, RMSSD is a well-known and frequently used HRV parameter, available on a variety of HRMs, HRV software programs and mobile applications, which could make it a suitable tool for home monitoring of AF recurrence after treatment. However, if RMSSD calculations are not available, SDNN (the standard deviation of the RR intervals) and SD1 (nonlinear parameter, derived from Poincaré plot), other frequently used HRV parameters and inter-related with RMSSD, might also be useful to detect AF [20].

Correct QRS detection is important to obtain correct RR intervals but the algorithms used by devices is often unknown. Filtering of data is often used to eliminate erroneously detected artifacts from the dataset before RMSSD is calculated. Several methods to record RR intervals were used in this study. Automatic analysis followed by manual correction of identified QRS complexes of an ECG trace using adhesive electrodes and calculation of RMSSD by specialized software without filtering was used as gold standard. However, this requires specialized equipment, expertise for ECG interpretation and data handling (exporting, importing), which is time consuming and expensive. Automatic analysis of the ECG trace by dedicated software reduces analysis time, but also introduces error. HRMs are increasingly being used by both veterinarians and horse owners, because they are relatively cheap and easy to use. They automatically detect heart rate and some devices also generate HRV calculations, including RMSSD. Previous studies assessing precision and accuracy of HRMs both in horses and in other animals, however, reported variable results and many HRMs have not been thoroughly validated in horses [23, 25–28]. Furthermore, different HRMs with different electrode design and RR detection software are available. Although in our study the ECG and HRM recordings were started exactly at the same time, the HRM recording appeared to show a lag and therefore our data did not allow to evaluate the performance of HRMs in correctly detecting individual QRS complexes. Since large differences in RMSSD between horses in AF and in SR have been shown [20], we hypothesized that, even if the use of a HRM introduced some error in RR detection, differences were sufficiently large to allow for reliable differentiation.

For the data obtained by the HRM, we used Polar Flow commercial software for HRV analysis. There are, however, numerous other, often cheaper, software programs and mobile phone applications available automatically importing RR data from HRMs via Bluetooth, displaying them in real time and immediately generating some basic HRV calculations. These applications might be useful candidates for home monitoring of horses at risk for AF recurrence but need to be validated.

Different HRV software programs might apply a different artifact correction method. The second goal of this study was to assess the effect of artifact correction levels on RMSSD as a discriminatory parameter for the AF detection. Artifact filters eliminate outliers, i.e. RR intervals that strongly differ from other RR intervals, by deleting them or replacing them by interpolated values. Although this filtering helps to delete artifacts, it will also blunt beat-to-beat variation which is typical for AF. Low level artifact correction resulted in improved sensitivity and specificity approaching 100% at all paces. During SR, second degree atrioventricular block (AVB) is a common finding in horses at rest with a large impact on HRV [29]. In our study, low level artifact correction probably eliminated most AVB from the analysis, since AVB leads to a very large increase in RR interval, and may be important in distinguishing AF from SR with frequent AVB. The Polar Flow software also uses an algorithm for artifact correction, which cannot be adapted. Unfortunately the authors were not able to obtain the details of this algorithm, but mean Polar values were close to mean Kubios values after Low to Moderate filtering. We have chosen to use the Kubios software because it is often used in human and veterinary medicine, it is free online available, and it allows to apply different filter levels. Our data suggest that low level filtering is needed, but that higher filter levels decrease specificity and sensitivity. We tested a limited number of filters only and cannot conclude upon the optimal filter. A large number of HRV analysis software programs are commercially available but one should be aware that each of them uses its own, usually unknown, QRS detection algorithm and type of filtering, which may have a big effect on the obtained RMSSD value.

In human medicine, artifact correction algorithms are also used in AF detection devices [16, 17, 30]. Reported sensitivity and specificity of these human devices range from 86.6–100% and 84.3–99.9%, respectively [15, 18, 19, 30].

Cut off values determined in this study were chosen to optimize sensitivity whilst maintaining good specificity. Because early diagnosis positively affects outcome and AF may occasionally be associated with dangerous ventricular tachyarrhythmias, our focus was to identify all horses with AF, even if this potentially included some false positives. False positives, however, will most likely arise from either incorrect RR registration, electrical interference and movement artifacts, or from horses with frequent arrhythmias other than AF. If these horses remain positive at consecutive HRM sessions during walk and trot, further electrocardiographic examination is indicated as other arrhythmias might be present.

Despite the relatively small study population and the unknown level of error introduced by the heart rate detection method of the HRM, differences in HRV between SR and AF were large, leading to reliable cut off values. Also, power calculations based upon RMSSD differences between horses in AF and in SR from a previous study [20], indicated sufficient power to detect significant differences with 5–7 horses. An important limitation of the study was its self-control design. We used horses before and after cardioversion since our goal was to differentiate AF from SR after successful treatment. Atrial premature depolarizations were often found post-cardioversion in these horses which could potentially make differentiation more challenging than if a control group of normal horses had been used. In this study we did not specify the number of arrhythmias but our previous study already showed that a high number of AVB during SR has an important effect on RMSSD, while a high number of atrial premature depolarization has not. We did not include horses with other arrhythmias than AVB and atrial premature depolarizations in this study and it remains to be proven whether this technique can also be used to diagnose AF in a random population of horses.

## Conclusions

We conclude that in horses before and after TVEC, AF can be differentiated from SR using RMSSD values obtained from automatically analyzed ECGs but also from an equine HRM. One should be aware that heart rate detection technique as well as level of artifact correction are important. Since we did not include horses with other arrhythmias in our study population, further studies are required before this technique can be used to differentiate AF from SR in a random population of horses.

## Additional file

Additional file 1: Receiver Operating Curve (ROC) for the use of RMSSD to distinguish atrial fibrillation from sinus rhythm in 14 warmblood horses during a 2-min heart rate recording at rest, (A), walk (B) and trot (C). ECGman: RR intervals obtained from a surface ECG after manual correction of QRS detection, using a commercial ECG software program (Televet 100 software version 5.1.2, Engel Engineering Services GmbH, Heusenstamm, Germany) to calculate RMSSD within a commercial software program (Kubios Heart Rate Variability Analysis Software, Varsitie 22, 70,150 Kuopio, Finland). ECGaut: RR intervals after automatic analysis of the surface ECG by a commercial ECG software program (Televet 100) to calculate RMSSD within a commercial software program (Kubios Heart Rate Variability Analysis Software). Five different levels of artifact correction were applied for ECGaut, going from a very low to a very high level of filtering. HRM: RR intervals obtained from a heart rate monitor (Polar heart rate sensor H7, Polar Electro Benelux, Dendermonde, Belgium) using commercial plastic electrodes (Polar equine heart rate electrode set, Polar Electro Benelux, Dendermonde, Belgium) to calculate RMSSD within the manufacturer’s software program (Polar Flow Software, Polar Electro Benelux, Dendermonde, Belgium). (TIF 636 kb)

## Abbreviations

AF: Atrial fibrillation
AUC: Area under the curve
AVB: Atrioventricular block
HRM: Heart rate monitor
HRV: Heart rate variability
RMSSD: Root mean squared successive differences in RR interval
ROC: Receiver operating curves
SD: Standard deviation
SR: Sinus rhythm
